# Identification of candidate genes conferring tolerance to aluminum stress in *Pinus massoniana* inoculated with ectomycorrhizal fungus

**DOI:** 10.1186/s12870-020-02719-3

**Published:** 2020-11-16

**Authors:** Haiyan Liu, Houying Chen, Guijie Ding, Kuaifen Li, Qifei Ren

**Affiliations:** 1 College of Forestry, Guizhou University/Institute for Forest Resources & Environment of Guizhou, Guiyang, 550025 Guizhou China; 2 Guizhou Botanical Garden, Guiyang, 550004 Guizhou China

**Keywords:** Aluminum toxicity, Afforestation, Gene expression, Ectomycorrhizal fungus, *Pinus massoniana*

## Abstract

**Background:**

*Pinus massoniana* Lamb. is an important afforestation tree species with high economic, ecological and medicinal values. Aluminum (Al) toxicity driven by soil acidification causes dieback of *P. massoniana* plantations. Previous studies showed that ectomycorrhizal fungi alleviate Al stress damages in *Pinus*, but the underlying molecular mechanisms and key genes induced by ectomycorrhizal fungi inoculation under Al stress in *Pinus* have not been explored. Herein, we applied Al stress for 60 days to *P. massoniana* seedlings inoculated with *Suillus luteus* (SL) and those non-inoculated. Then, we compared their growth parameters and transcriptome in order to detect candidate genes induced by SL conferring Al tolerance in *P. massoniana*.

**Result:**

Our results showed that SL inoculation confers Al stress tolerance in *P. massoniana* through improved growth performance, strong antioxidant enzyme activities and reduced malondialdehyde accumulation as compared to non-inoculated seedlings. Transcriptome sequencing further supported these findings as very few genes (51 genes) were transcriptionally altered by Al in SL inoculated plants as compared to non-inoculated plants (2140 genes). We identified three core genes (*cox1*, *cox3* and *Nd1*) that were strongly up-regulated by Al in the SL inoculated plants but were down-regulated in the non-inoculated plants. We also identified 42 genes specifically regulated by SL inoculated plants under Al stress, which are involved in a wide range of biological processes such as antioxidative response, transporters, hormone signaling and plant pathogen infection responses.

**Conclusions:**

Altogether, our data suggest that SL inoculation induces priming of key stress response pathways and triggers specific genes that efficiently alleviate Al stress effects in *P. massoniana*. The candidate genes resources generated in this study are of utmost importance for functional characterization and molecular studies aiming at improving Al tolerance in plants.

**Supplementary Information:**

The online version contains supplementary material available at 10.1186/s12870-020-02719-3.

## Background

*Pinus massoniana* Lamb. is an important afforestation tree species belonging to the Pinaceae family. It is native to southern China and is one of the dominant species for forest plantations in China [1]. *P. massoniana* is a pioneer species, highly tolerant to environmental stresses and grows well in barren areas and metal-contaminated soils [2–6]. It not only contributes to meeting the growing demand of wood products but also reduces pressures on natural forests and significantly contributes to restoration of degraded soils [7–10]. Besides these economic and ecological values, several studies have demonstrated the pharmacological properties of *P. massoniana* bark and needles for the treatment of rheumatism, intestinal parasites, hypertension, neurasthenia, skin complaints and cancer [11–14].

*Pinus* species, including *P. massoniana*, are natural hosts for diverse ectomycorrhizal fungal species [15–19]. It has been reported that ectomycorrhizal symbiosis establishment in the root system confers improved growth performance and tolerance to biotic and abiotic stress in host plants [20–22]. This has been ascribed to the improved nutrient and water acquisition, photosynthetic rate and enhancement of the antioxidant systems and immune system in the hosts [23, 24]. In *Pinus*, previous studies have shown that ectomycorrhizal fungi improve plant growth and tolerance to drought stress, salinity stress, low-phosphorous stress, heavy metals toxicity, etc. [4, 6, 25–28].

Aluminum (Al) toxicity driven by soil acidification is a long-lasting problem causing forest dieback in many regions of the world [29], particularly in China where natural and forest plantations are declining [30]. Acid rain and anthropogenic soil acidification caused by long-range air pollution and intensive uses of acid-forming fertilizers create nutrient depletion in the soil and accelerate bio-availability of toxic elements such as Al^3+^ [31–33]. Von Uexkuell and Mutert estimated early in 1995 that acid soils cover more than 70% of potential arable soils [34] but this value could be higher nowadays with the ever-growing industrialization and intensive agriculture. High amount of Al in the soil inhibits plant root growth and decreases nutrient and water uptake [35, 36], leading to significant reductions of plant productivity. It also increases the levels of reactive oxygen species (ROS), leading to lipid peroxidation and cell death [37]. Deciphering the mechanisms of Al tolerance in plants has catalyzed numerous studies and our understanding on the topic is increasing. For example, the exclusion and internal detoxification of Al have been demonstrated in several plants [38–40] and some related genes such as the aluminum activated malate transporter (ALMT) and multidrug and toxic compound extrusion (MATE) have been discovered [40–45].

In forest tree species, some degrees of tolerance to Al toxicity have been observed with a variation among species and genotypes [46–48]. For example, *Betula pendula* is able to tolerate Al concentration up to 3 mM [49], while Liu and Liu [47] found that the lowest concentration of aluminum toxicity in *P. massoniana* was 0.15 mM. Besides the intrinsic capacity to exclude Al from root of each plant species, ectomycorrhizal symbiosis establishment in the root system can provide another layer of defensive force against Al stress damages to the host plant. In support to this idea, previous studies have shown that ectomycorrhizal fungi alleviate Al stress damages in *Pinus* [50, 51]. However, the underlying molecular mechanisms and key genes induced by ectomycorrhizal fungi inoculation under Al stress in *Pinus* have not been explored.

In a preliminary experiment, we found that inoculation with the ectomycorrhizal fungus species *Suillus luteus* (SL) promotes *P. massoniana* growth and imparts Al stress tolerance. We therefore designed the present study to explore the transcriptome of SL inoculated plants and non-inoculated plants under Al stress in order to detect candidate genes mediated by SL conferring Al stress tolerance in *P. massoniana*.

## Result

### Morpho-biochemical responses to aluminum stress with or without *ectomycorrhizal fungus* inoculation

**Fig. 1:**
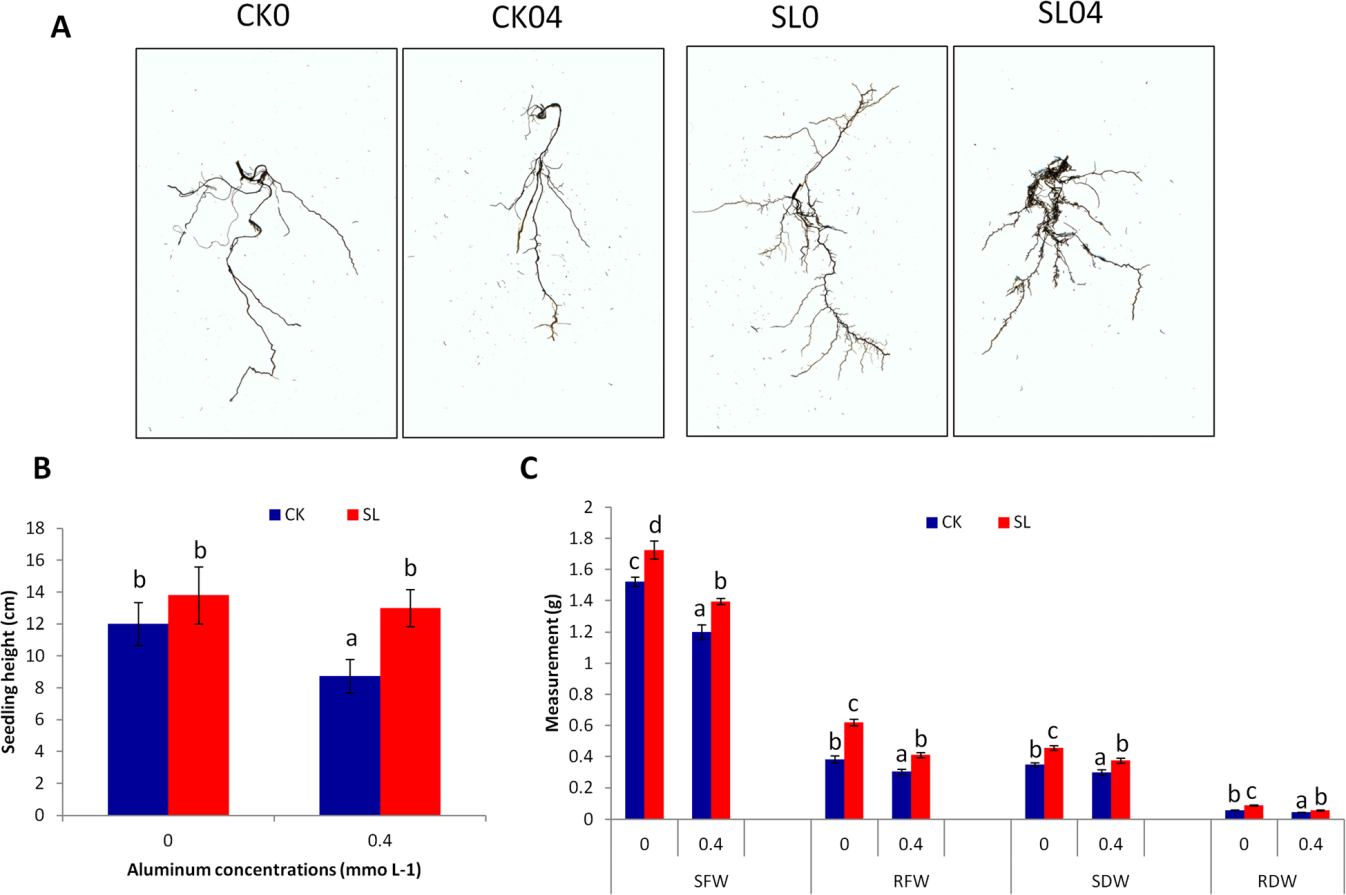
Morphological parameters of *Pinus massoniana* inoculated with *Suillus luteus* (SL) and non-inoculated plants (CK) under Al stress. **a** Scans of the root samples from *SL* inoculated plants and CK plants under 0 and 0.4 mmol L^− 1^ Al, **b** seedling height, **c** shoot fresh weight (SFW), root fresh weight (RFW), shoot dry weight (SDW), root dry weight (RDW). Bars represent average values ± SD of 10 replicates. Different letters above bars indicate significant difference at *P* < 0.05

*P. massoniana* seedlings were subjected to Aluminum (Al) stress for 60 days with *Suillus luteus* (SL) inoculation or without inoculation (CK). Several morpho-biochemical parameters were investigated. As shown in Fig. 1a, the root density of the seedlings inoculated with the ectomycorrhizal fungus (SL) was higher as compared to non-inoculated seedlings (CK) independently of the Al concentrations, demonstrating the successful inoculation in our experiment. Quantitative analysis of root traits showed that Al treatment reduced all root traits. However, SL inoculation improved the root surface area, average root diameter and root number traits under Al treatment as compared to CK plants (Table 1). Without Al, the seedling height was slightly increased in SL inoculated plants as compared to CK but not significantly (Fig. 1b**)**. Other traits such as shoot fresh weight (SFW), root fresh weight (RFW), shoot dry weight (SDW), root dry weight (RDW) were significantly improved by SL inoculation (Fig. 1c). With 0.4 mmol L^− 1^ Al application, all morphological traits were reduced independently of SL inoculation (Fig. 1b, c), indicating that Al affects *P. massoniana* seedling growth. Noteworthy, all morphological traits were significantly higher in SL inoculated plants than in CK plants, implying that SL improves *P. massoniana* tolerance to Al stress (Fig. 1b, c).

**Table 1:** Characterization of root traits in *Pinus* seedlings. Different letters display significant difference at *P* < 0.05

Samples	Main root length (cm)	Root surface area (cm^**2**^)	Average root diameter (mm)	Root number
CK0	6.24 ± 1.41a	0.73 ± 0.8a	0.36 ± 0.10b	50.75 ± 8.04b
CK04	3.98 ± 0.80b	0.39 ± 0.12c	0.20 ± 0.05c	30.00 ± 8.11c
SL0	5.41 ± 1.30a	0.77 ± 0.08a	0.55 ± 0.07a	63.25 ± 11.45a
SL04	3.64 ± 0.70b	0.52 ± 0.15b	0.39 ± 0.05b	47.00 ± 9.90b

**Fig. 2:**
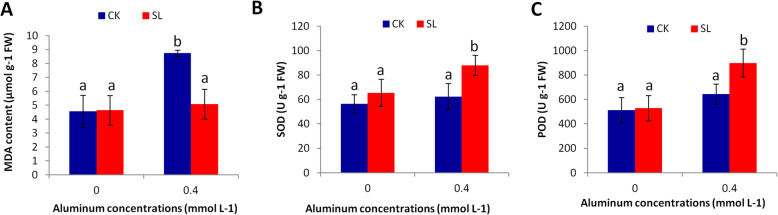
Enzyme activities and malondialdehyde (MDA) content in *Pinus massoniana* inoculated with *Suillus luteus* (SL) and non-inoculated plants (CK) under Al stress. **a** MDA content, **b** Superoxide dismutase (SOD) activity, **c**, peroxidase (POD) activity in plants grown under 0 and 0.4 mmol L^− 1^ Al. Bars represent average values ± SD of 10 replicates. Different letters above bars indicate significant difference at *P* < 0.05

Under stress conditions, plants accumulate excessive levels of reactive oxygen species (ROS). Malondialdehyde (MDA) content is associated with lipid peroxidation via an increased generation of ROS [52]. A high level of MDA is an indicator of a high level of stress damage. We measured the MDA content in needle samples from the four treatments (Fig. 2a). Without Al stress, similar levels of MDA could be observed in CK and SL inoculated plants. However under Al stress, although MDA content increased in both CK and SL inoculated plants, a significantly higher MDA content was measured in CK plants compared to SL inoculated plants. This result suggests that CK plants suffered from oxidative damages under Al stress while SL inoculation helped to keep MDA level in a normal range.

To effectively combat ROS excessive accumulation in plants, a strong activation of antioxidant enzymes such as superoxide dismutase (SOD) and peroxidase (POD) is essential [53]. In this study, we observed that both CK and SL inoculated plants increased their SOD and POD activities under Al stress. However, significantly higher SOD and POD activities were noticed in SL inoculated plants as compared to CK plants (Fig. 2b, c). Altogether, our results suggest that SL inoculation enhances Al stress tolerance in *P. massoniana* through enhanced antioxidant enzymes activity.

### Portray of the transcriptome sequencing and assembly

**Fig. 3:**
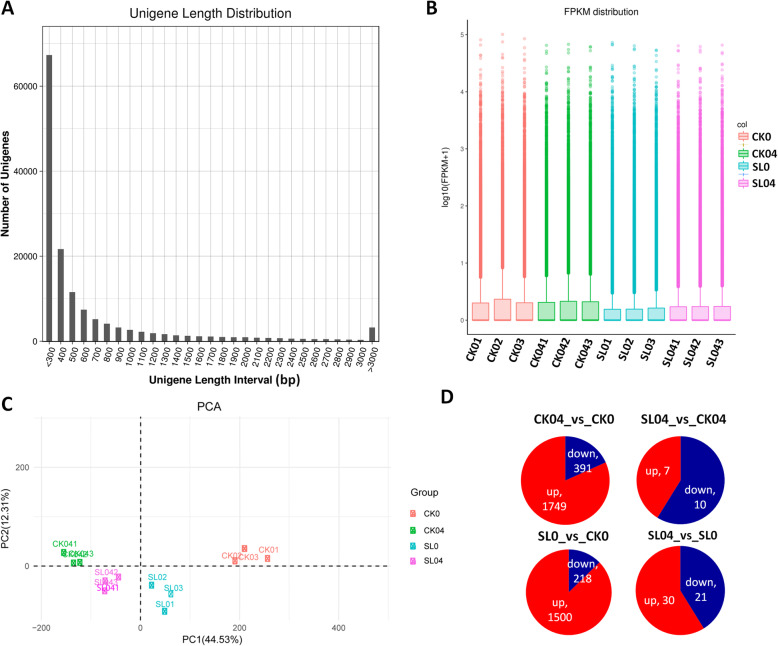
Overview of the transcriptome sequencing and quality. **a** Unigene length distribution, **b** Boxplots displaying overall distribution of gene expression among samples, **c** Principle component analysis of expressed genes, **d** Pie chart showing the number of up- and down-regulated genes in each compared group. SL0, SL04, CK0 and CK04 represent the *Suillus luteus* (SL) inoculated plants without Al stress application, SL inoculated plants with 0.4 mmol L^− 1^ Al stress application, plants non-inoculated without Al stress application and plants non-inoculated with 0.4 mmol L^− 1^ Al stress application, respectively

In order to get insight into the molecular basis of Al stress tolerance induced by SL inoculation in *P. massoniana*, we sequenced the transcriptome of needle samples from the four treatments. With three replicates in each treatment, 12 samples in total were sequenced, yielding on average 54,226,624 raw reads per sample (Table 2). After cleaning, we obtained 89 Gb data with Q30 quality score higher than 93% and error rate lower than 0.03. The clean data were de novo assembled as the reference gene set using the Trinity software and 145,434 unigenes spanning 90,173,863 bp long were obtained with a mean length of 620 bp and a N50 of 1022 bp long. The unigene length distribution is shown in Fig. 3a. The unigenes were annotated in five different databases including NR, SwissProt, PFAM, GO and KO, with 67% of the total unigenes annotated in at least one database (Table 3).

**Table 2:** Statistics of the transcriptome sequencing and quality check

Samples	Raw_reads	Clean_reads	Clean_bases (Gb)	Error (%)	Q20 (%)	Q30 (%)	GC (%)
CK01	50,686,474	50,295,380	6.99	0.02	97.87	93.76	47.07
CK02	50,394,888	50,015,274	6.94	0.02	97.95	93.92	47.14
CK03	61,303,972	60,958,750	8.43	0.029	97.99	94.03	47.34
CK041	50,440,018	50,157,384	6.86	0.029	97.96	94	47.7
CK042	57,788,340	57,453,616	7.9	0.029	97.87	93.75	47.58
CK043	58,747,552	58,404,162	8.02	0.029	97.87	93.76	47.68
SL01	57,381,552	57,042,586	7.8	0.029	97.99	94.08	47.95
SL02	63,618,448	63,275,858	8.73	0.029	97.95	93.96	47.37
SL03	59,962,230	59,628,096	8.24	0.029	97.96	93.93	47.35
SL041	43,256,016	42,968,462	5.96	0.029	97.84	93.67	46.98
SL042	46,580,296	46,369,104	6.43	0.02	98.15	94.34	47.03
SL043	50,559,704	50,299,706	6.98	0.02	98.06	94.19	47.03

**Table 3:** Statistics of the unigene annotation

Database	Number of unigenes	Percentage (%)
Annotated in NR	56,449	38.81
Annotated in SwissProt	35,420	24.35
Annotated in PFAM	28,256	19.43
Annotated in GO	26,522	18.24
Annotated in KEGG	11,912	8.19
Annotated in all Databases	137	0.09
Annotated in at least one Database	97,440	67
Total Unigenes	145,434	100

The gene expression was estimated with fragments per kilobase of exon per million fragments mapped (FPKM) and FPKM value > 1 was used as a threshold to determine the expressed genes (Fig. 3b). Using the FPKM data, we performed a principal component analysis (PCA) to check the clustering pattern of the samples from the four treatments and their replicates. As shown in Fig. 3c, PC1 and PC2 together contributed to over 66% of the global variation. PC1 clearly separated Al treated samples and the non treated samples. By PC2, we could observe a separation between SL inoculated samples from the non-inoculated samples. In addition, the replicates of each treatment were found closely clustered, showing that the quality of the transcriptome sequencing was good enough to proceed to further analyses.

### Differentially expressed genes

In order to identify the differentially expressed genes (DEG), we cross-compared the gene count between treatments using following screening criteria: |log2 fold change| ≥ 2 [54], and false discovery rate (FDR) correction set at *P* < 0.05. In total, 2140, 1718, 17 and 51 DEGs were detected in CK04_vs_CK0, SL0_vs_CK0, SL04_vs_CK04 and SL04_vs_SL0, respectively (Fig. 3d). Gene ontology enrichment analysis of the DEGs showed various biological pathways (metabolic process, photosynthesis, oxido-reduction process, nucleotide binding, etc.) affected by Al and SL treatments (Figure S1). Kyoto Encyclopedia of Genes and Genomes enrichment analyses of the DEGs showed that phorphyrin and chlorophyll metabolism, zeatin biosynthesis were the most enriched pathways affected by Al and SL treatments (Figure S2). The comparison CK04_vs_CK0 provides DEGs involved in Al stress response without SL inoculation, while SL04_vs_SL0 provides DEGs involved in Al stress response with SL inoculation. The conspicuous difference in the number of DEGs between these two comparisons (2140 vs 51) indicates that fewer genes were transcriptionally altered by Al under SL inoculation as compared to conditions without SL inoculation. This confirms that SL inoculation confers Al stress tolerance in *P. massoniana*.

### Core conserved genes regulated by Al stress independently of SL inoculation

**Fig. 4:**
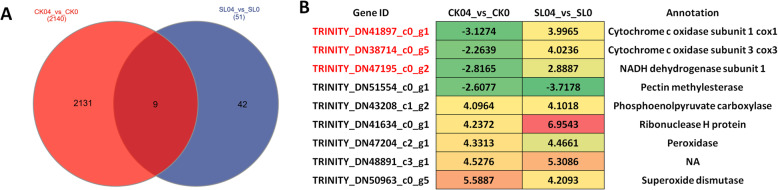
Identification of candidate genes imparting Al stress tolerance in *Suillus luteus* (SL) inoculated *Pinus massoniana* seedlings. **a** Venn diagram depicting the number of shared and specific differentially expressed genes (DEGs) induced by Al without SL inoculation (CK04_vs_CK0) and with SL inoculation (SL04_vs_SL0). **b** Heatmap showing the log2 fold change of nine core DEGs between CK04_vs_CK0 and SL04_vs_SL0. Gene names highlighted in red are those with an opposite pattern of regulation between the two groups. SL0, SL04, CK0 and CK04 represent the SL inoculated plants without Al stress application, SL inoculated plants with 0.4 mmol L^− 1^ Al stress application, plants non-inoculated without Al stress application and plants non-inoculated with 0.4 mmol L^− 1^ Al stress application, respectively

We compared the DEGs induced by Al without SL inoculation (CK04_vs_CK0) and with SL inoculation (SL04_vs_SL0) in order to identify the core regulated DEGs altered by Al stress. As shown in Fig. 4a, nine core DEGs were detected of which, six DEGs (*TRINITY_DN51554_c0_g1*, *TRINITY_DN43208_c1_g2*, *TRINITY_DN41634_c0_g1*, *TRINITY_DN47204_c2_g1*, *TRINITY_DN48891_c3_g1*, *TRINITY_DN50963_c0_g5*) displayed similar patterns of regulation between CK04_vs_CK0 and SL04_vs_SL0 (Fig. 4b), indicating that these genes are essential for Al response in *P. massoniana.* The gene *TRINITY_DN48891_c3_g1* was not functionally annotated, implying it may be an Al-responsive gene specific to *P. massoniana*. In contrast, we identified three other genes (*TRINITY_DN41897_c0_g1* (*cox1*), *TRINITY_DN38714_c0_g5* (*cox3*) and *TRINITY_DN47195_c0_g2* (*Nd1*)), which displayed opposite patterns of regulation between CK04_vs_CK0 and SL04_vs_SL0 (Fig. 4b). The strong up-regulation of these genes in SL04_vs_SL0 may indicate a tolerance mechanism, which the control plants failed to trigger under Al stress (CK04_vs_CK0).

### Candidate genes conferring Al stress tolerance induced by SL inoculation

Since *P. massoniana* seedlings tolerate well Al stress under SL inoculation, we considered the specifically regulated genes in SL04_vs_SL0 as candidate genes imparting Al stress tolerance. In total, 42 DEGs were identified in SL04_vs_SL0 (Fig. 4a**,** Table 4) but they were not significantly altered in the non-inoculated plants under Al stress (CK04_vs_CK0). Most of the detected genes are involved in antioxidative response, hormone signaling and more importantly plant pathogen infection responses. This shows that prior to exposure to Al stress, SL inoculated plants have an enhanced immune system and ROS scavenging machinery, which may be instrumental for promptly responding to Al stress. Besides, several unannotated DEGs were detected, representing interesting gene resources for future functional characterization.

**Table 4:** Genes specifically regulated in SL inoculated plants in response to Al stress

Gene ID	Log2 Fold Change SL04/SL0	Annotation
*TRINITY_DN50947_c2_g1*	−10.045	pre-mRNA-processing protein 40C-like
*TRINITY_DN42182_c1_g1*	−6.626	NA
*TRINITY_DN41191_c0_g1*	−6.3241	Pectin methylesterase (*PME*)
*TRINITY_DN40212_c0_g1*	−5.8947	NA
*TRINITY_DN36359_c1_g1*	−5.8146	Olee1-like protein
*TRINITY_DN36491_c0_g1*	−5.6695	NA
*TRINITY_DN40533_c0_g1*	−4.9629	DUF538
*TRINITY_DN47888_c0_g1*	−4.792	DUF3799
*TRINITY_DN44356_c0_g1*	−3.6987	Pathogenesis-related protein
*TRINITY_DN53237_c4_g1*	−2.957	Subtilisin-like protease SBT5.6
*TRINITY_DN10972_c0_g1*	−2.5905	NA
*TRINITY_DN44990_c0_g2*	−2.5865	Ubiquitin
*TRINITY_DN49726_c1_g1*	−2.4029	C2 domain
*TRINITY_DN49726_c1_g2*	−2.3392	NA
*TRINITY_DN44727_c0_g4*	−2.2457	UDP-rhamnose:rhamnosyltransferase 1
*TRINITY_DN44741_c1_g1*	−2.1912	NA
*TRINITY_DN50117_c0_g5*	−2.1671	Downy mildew resistant 6 oxygenase
*TRINITY_DN47573_c2_g4*	−2.0719	NA
*TRINITY_DN46639_c1_g3*	−2.0478	NA
*TRINITY_DN50628_c0_g3*	−2.0341	DUF674
*TRINITY_DN47580_c3_g1*	2.0214	Protein phosphatase 2C
*TRINITY_DN40477_c0_g2*	2.1501	Superoxide dismutase (*SOD*)
*TRINITY_DN52128_c2_g4*	2.2455	Chaperone protein ClpB1-like protein
*TRINITY_DN48901_c0_g1*	2.3314	60S acidic ribosomal protein P0
*TRINITY_DN46679_c1_g3*	2.3589	NA
*TRINITY_DN49138_c0_g1*	2.5076	Plant protein 1589 of unknown function (A_thal_3526)
*TRINITY_DN45082_c2_g2*	2.6635	NA
*TRINITY_DN47946_c1_g6*	3.0243	NA
*TRINITY_DN50109_c1_g2*	3.131	NA
*TRINITY_DN51142_c2_g1*	3.2377	Serine carboxypeptidase-like 40
*TRINITY_DN48446_c0_g1*	3.299	Peroxidase (*POD*)
*TRINITY_DN51077_c0_g4*	3.3457	LRR receptor-like serine/threonine-protein kinase
*TRINITY_DN38876_c0_g1*	3.4286	NA
*TRINITY_DN50590_c0_g1*	3.5695	Superoxide dismutase (*SOD*)
*TRINITY_DN49159_c3_g1*	4.3167	Indole-3-acetic acid (*IAA*)
*TRINITY_DN47375_c2_g1*	4.7598	NBS-LRR protein G6229
*TRINITY_DN49087_c1_g2*	4.8788	TIR-NBS-LRR protein
*TRINITY_DN39211_c0_g1*	7.2334	Multidrug and toxic compound extrusion (*MATE*)
*TRINITY_DN45197_c0_g1*	7.3014	Gluthatione S transferase (*GST*)
*TRINITY_DN47067_c5_g1*	8.1155	Peroxidase (*POD*)
*TRINITY_DN49617_c1_g1*	10.6606	Chaperone protein ClpB1
*TRINITY_DN44453_c0_g2*	21.5957	NA

### qRT-PCR validation of selected genes

**Fig. 5:**
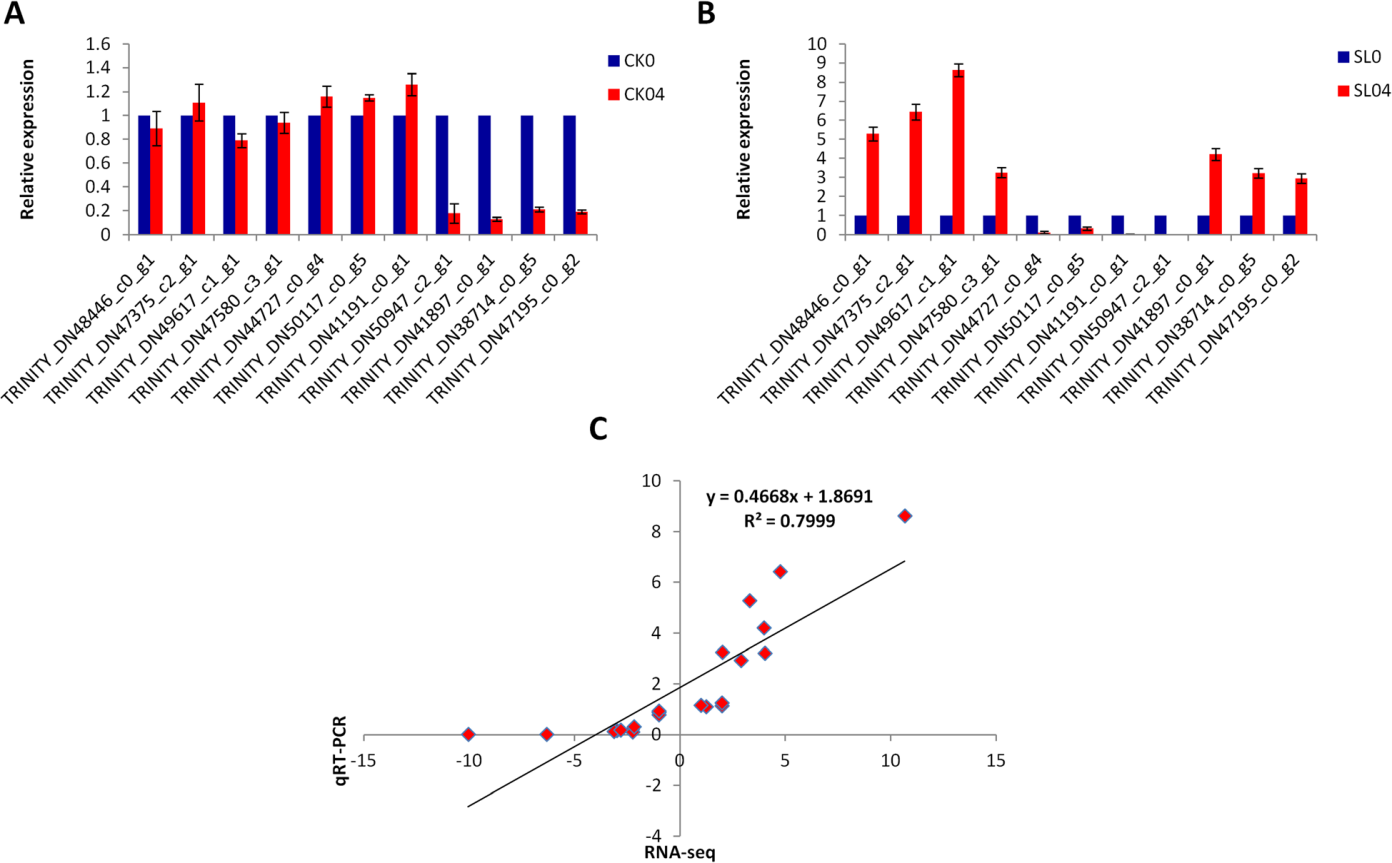
qRT-PCR validation of selected candidate genes. **a** non-inoculated plants, **b** inoculated plants. The x-axis represents the genes while the y-axis represents the relative expression of each gene. The bars show standard deviation. **c** Pearson correlation between qRT-PCR and RNA-seq data. SL0, SL04, CK0 and CK04 represent the *Suillus luteus* (SL) inoculated plants without Al stress application, SL inoculated plants with 0.4 mmol L^− 1^ Al stress application, plants non-inoculated without Al stress application and plants non-inoculated with 0.4 mmol L^− 1^ Al stress application, respectively

We selected 11 various candidate genes involved in Al stress response in *P. massoniana* to validate their expression levels using qRT-PCR. The gene *Actin2* was used as internal control for expression normalization. The qRT-PCR result showed that the expression levels of all selected genes were significantly altered by Al stress (Fig. 5). In addition, qRT-PCR results were strongly correlated with the RNA-seq report (R^2^ = 0.8), indicating that the interpretation of the RNA-seq report in this study is reliable.

## Discussion

### *Suillus luteus* inoculation confers aluminum stress tolerance in *P. massoniana* seedlings

By applying Aluminum (Al) concentrations higher than 0.1 mM, Liu and Liu [47] found that *P. massoniana* growth is significantly reduced. In this study, we observed a significant inhibition of *P. massoniana* growth under 0.4 mM Al (Fig. 1; Table 1). Since high Al concentrations (> 1 mM) were reported in forest soils in southern China [30, 55], it is evident that *P. massoniana* suffers from Al toxicity in the field. Various ectomycorrhizal fungi have been shown to improve *Pinus* growth performance and tolerance to diverse biotic and abiotic stresses, including Al toxicity [4, 6, 25–28]. *Suillus luteus* (SL) is an ectomycorrhizal fungus widely found in *Pinus* plantations [56, 57]. It has been reported that SL can grow in adverse soil conditions such as soils with high salinity, water scarcity, high Mg, Zn, Cd, Pb, Ni, Al [6, 50, 51, 56, 58, 59]. In particular, numerous studies have demonstrated that SL is an Al tolerant fungal species [60–63] and it can facilitate the regeneration and plantation of *Pinus* seedlings in Al contaminated areas. The results of this study fully corroborate previous findings because our SL inoculated *P. massoniana* plants tolerated well Al stress (Fig. 1). Yamamoto et al. [37] revealed that high amount of Al induces excessive reactive oxygen species (ROS) accumulation in cells, which ultimately leads to cell death. The weak malondialdehyde content coupled with strong antioxidant enzymes activity in SL inoculated plants (Fig. 2) suggests that SL limits ROS related damages in *P. massoniana*. This was further confirmed by the significantly lower number of DEGs found in SL inoculated plants as compared to non-inoculated plants under Al stress (Fig. 3d) [64, 65]. In future studies, it will be important to clarify the physiological mechanisms of Al tolerance induced by SL inoculation in *P. massoniana*. For example, by determining Al content in various plant tissues before and after Al treatment with and without SL inoculation, we will be able to understand how SL affects Al uptake by *P. massoniana*.

### New gene resources for improving Al tolerance in *P. massoniana*

The mechanisms of Al response including, reduced Al uptake and detoxification of absorbed Al in the symplasm have been well studied in plants [66]. Several related genes with various biological functions such as stress response, membrane transporter, organic acid metabolism, cell wall modification, signaling, hormones, transcription factors have been identified [45, 66–69]. In this study, we identified nine core genes playing diverse functions in response to Al stress in *P. massoniana* independently of SL inoculation (Fig. 4). The up-regulation of the genes *TRINITY_DN47204_c2_g1* (SOD) and *TRINITY_DN50963_c0_g5* (POD) showed that activation of antioxidant enzymes is a basic response to Al stress in *P. massoniana*. The secretion of organic acids such as citrate, malate and oxalate from the roots detoxifies soil Al [70]. The strong up-regulation of the gene *TRINITY_DN43208_c1_g2* (phosphoenolpyruvate carboxylase) which is involved in oxalate metabolism suggests that this mechanism is conserved in *P. massoniana* as demonstrated in other plant species [71, 72]. We noticed the down-regulation of a pectin methylesterase gene (PME, *TRINITY_DN51554_c0_g1*) which is known to be involved in cell wall structure and high level of PME correlates with high levels of Al adsorption. Therefore, *P. massoniana* tends to reduce Al adsorption by repressing the expression of the *PME* [72, 73]. Three core Al responsive genes (Cytochrome c oxidase (*cox*)1, *cox3* and NADH dehydrogenase subunit 1 (*Nad1*)) associated with mitochondrial activity displayed opposite patterns between SL inoculated and non-inoculated plants. It is well documented that dysfunction of mitochondrial activity seriously impacts on plant fitness [74, 75] and Yamamoto et al. [76] evidenced that Al stress disrupts mitochondrial functions and provokes high accumulation of ROS in tobacco and pea. In this study, all these genes were up-regulated in SL inoculated plants while a strong down-regulation was observed in non-inoculated plants. We deduced that Al stress-induced shut down of mitochondrial activity may have led to high ROS accumulation and subsequently to the growth inhibition observed in non-inoculated plants. We propose these three genes, pending further validation, as marker genes for screening Al tolerant *Pinus* genotypes.

When engaged in symbiosis with tolerant ectomycorrhizal fungi, plant responses to Al are ameliorated since fungi can immobilize toxic Al into the mycorrhizal roots and surrounding soil environment and improve plant mineral nutrition to ensure normal growth [77]. However, genes mediated by ectomycorrhizal fungi in response to Al stress in host plants have been poorly investigated [23]. In this study, we identified 42 candidate genes unique to SL inoculated plants that may confer Al stress tolerance (Table 4). Most of these genes are involved in Al well known responsive pathways such as antioxidative response, hormone signaling, transporters and plant pathogen infection responses [66]. For example, peroxidase, gluthatione S transferase, multidrug and toxic compound extrusion, indole-3-acetic acid, superoxide dismutase, protein phosphatase 2C, pectin methylesterase have been reported to regulate Al response in plants [45, 54, 72, 73, 78, 79]. A major finding in this study was the numerous genes involved in defense and plant pathogen infection pathway (Table 4). Similar to our results, Luo et al. [23] reported that ectomycorrhizal fungi induced high number of defensive and pathogen infection metabolites and genes under salinity stress in poplar. They concluded that ectomycorrhizal fungi boost the host immune system by priming roots for increased salt tolerance. We speculate that a similar mechanism was established in SL inoculated *P. massoniana* plants in this study and it helped seedlings to promptly and efficiently combat Al stress effects.

## Conclusions

Inoculation of *P. massoniana* with the ectomycorrhizal fungus *Suillus luteus* (SL) improves seedling growth and confers Aluminum (Al) stress tolerance. Disruption of the mitochondrial functions through down-regulation of key genes such as *cox1*, *cox2* and *Nad1* is probably critical for Al inhibition of growth in non SL inoculated seedlings. Therefore, strategies to activate these genes under Al stress in *P. massoniana* should be further investigated. We also identified several candidate genes including some unannotated genes that may play cardinal roles in Al stress tolerance. Functional characterization of these gene resources will provide necessary tools for engineering Al tolerant *Pinus* plants with less dependence on ectomycorrhizal fungi.

## Method

### Plant material, ectomycorrhizal fungus and stress treatment

*P. massoniana* Lamb. was used as plant material in this study. The seeds were collected in November 2018 from a superior provenance tree (20 years) planted in Duyun City, Guizhou Province, China. The ectomycorrhizal fungus species *Suillus luteus* (SL), identified as an aggressive colonizer to *P. massoniana* [56], was used for plant inoculation. The fruiting part of the fungus was collected from was collected from *P. massoniana* pure plantation in Longli Forest Farm (N26°28′01″, E107°00′37″), Longli County, Guiyang City, Guizhou Province, China. The formal identification of the plant material and fungus was undertaken by the corresponding author of this article (Professor Guijie Ding). Plant material is available at the National base of *P. massoniana* (N26°169′ ~ 26°170′, E107°623′ ~ 107°624′) at Maanshan forest farm, Duyun City, Guiyang City, Guizhou Province and a voucher specimen has been deposited at Guizhou Botanical Garden, Guiyang, China, under the accession number: xgnk-2003-a12. No permission was necessary to collect such samples. Inoculum preparation and plant inoculation were performed following detailed descriptions in works of Yu et al. [6]. Seedlings were raised from February to July 2019 and 6-months old seedlings inoculated with *S. luteus* (inspected for successful mycorrhization) were used as test materials while 6-months old seedlings without inoculation were used as control.

The experiment was carried out in a greenhouse with light intensity of 600–800 μmol m^− 2^ s^− 1^, relative humidity of 55%, photoperiod of 16 h, 25 °C at light and 18 °C in the dark. The quartz sand was rinsed, sterilized in an autoclave (pressure 0.14 MPa, 121 °C) for 2 h, and then loaded into plastic pots (21 cm × 15 cm × 18.5 cm). Uniform seedlings were selected and transplanted into the pots. They were kept under normal growth conditions for 2 weeks by pouring frequently 1/2 Hoagland nutrient solution. Then, the stress treatment started. Four treatments were set, namely 2 inoculated treatments SL0 (0 mmol L^− 1^ Al^3+^), SL04 (0.4 mmol L^− 1^ Al^3+^) and 2 non-inoculated treatments CK0 (0 mmol L^− 1^ Al^3+^), CK04 (0.4 mmol L^− 1^ Al^3+^) following descriptions of Liu and Liu [47]. For Aluminum (Al) stress, Al was added to total Hoagland nutrient solution in the form of anhydrous AlCl_3_ and the pH value was adjusted to 4.1 ± 0.1 [47] with 0.1 M diluted HCl or NaOH to maintain acidic conditions. In order to maintain the Al activity, 0.5 mmol L^− 1^ CaCl_2_ was added into the nutrient solution at the same time to avoid interaction between Al ion and solution ions. The treatment solution was poured once a week. Each treatment was repeated five times containing 15 pots each, with three plants per pot. Plants were collected on the 60th day after the induction of Al stress to measure the growth and physiological parameters in the four groups. In addition, we randomly selected three plants from different replicates in each treatment and needle samples were collected for transcriptome analysis. Samples were frozen in liquid nitrogen and stored at − 80 °C for further use.

### Measurement of morphological parameters

Ten plants per treatment were selected from the five replicates (two plants per replicate), gently shaken to remove the quartz sand on the root surface, and carefully washed with running water. Root quantitative traits were measured from scanned images from a desktop scanner (EPSON Perfection V800 Photo, CA, USA) using the WinRHIZO Pro software (Regent Instruments Inc., Quebec, Canada). Seedling height was measured with a vernier caliper; the root and shoot fresh weights were recorded separately using an electric balance (Type: ML 204; Mettler Toledo Company, Greifensee, Switzerland; measurement accuracy 0.0001 g). Samples were dried at 80 °C to constant weight and the dry weights were recorded.

### Determination of SOD, POD, soluble sugar and MDA

The content of Malondialdehyde (MDA) and the activities of superoxide dismutase (SOD) and peroxidase (POD) in the needle samples from the four treatments were determined in five replicates separately according to the instructions provided by kits (COMIN, Keming Biotechnology Co. Ltd., Suzhou, China). The content of MDA was expressed as μmol g^− 1^ FW. SOD and POD activities were expressed as units per gram fresh weigh (U g^− 1^ FW). One unit (U) of SOD activity was defined as the activity of SOD when the inhibition of the xanthine oxidase coupling reaction system was 50%, while one unit (U) of POD activity was defined as the variation of 0.01 in A470 per mL reaction system.

### Statistical analysis

All data were statistically analyzed with Minitab18 software (*P* < 0. 05). Tukey’s test based on analysis of variance (ANOVA) was chosen to analyze the data.

### RNA extraction, cDNA library construction and transcriptome sequencing

Experiments are conducted following standard procedures of Shanghai Applied Protein Technology, Co., Ltd. (APT, Shanghai, China). Briefly, total RNAs were extracted from 12 needle samples with RNAprep Pure Plant Kit (TIANGEN, Beijing, China). Concentrations of the samples were quantified by a NanoDrop 2000C spectrophotometer (Thermo Fisher Scientific). To get high quality RNA, samples were tested on 1% agarose gel electrophoresis for the integrity of RNA and DNA contamination. For accurate detection of RNA integrity, Agilent 2100 Bioanalyzer was used. RNA quantification was performed using Qubit RNA Assay Kit in Qubit 2.0 Flurometer (Life Technologies, Carlsbad, CA, USA). Next, RNA integrity was checked by the RNA Nano 6000 Assay Kit of the Agilent Bioanalyzer 2100 system (Agilent Technologies, Santa Clara, CA, USA). For cDNA synthesis, a total 1 μg RNA for each sample was treated with DNase I to eradicate the genomic DNA and then used as a template for reverse transcription (QuantiTect Reverse Transcription Kit, Qiagen, China).

We added fragment buffer to break into short segments using short segment RNA as template. Sequencing libraries was created using NEB Next Ultra RNA Library Prep Kit following manufacturer′s instructions. The index codes were added to each sample. Briefly, the mRNA was purified from 3 μg total RNA of each of three replicate using poly-T oligo-attached magnetic beads and then broken into short fragments to synthesize first strand cDNA. The second strand cDNA synthesis was subsequently performed using DNA Polymerase I and RNase H. PCR was carried out with Phusion High Fidelity DNA polymerase using universal PCR primers and index (×) primer. Finally, 12 paired-end cDNA libraries with an insert size of 300 bp were constructed for transcriptome sequencing and sequenced on Illumina HiSeq 4000 platform (Illumina Inc., San Diego, USA) at Shanghai Applied Protein Technology, Co., Ltd. (APT, Shanghai, China).

### De novo assembly, functional annotation, expression analysis

The clean reads were obtained after trimming adapter sequences, removal of low quality (containing > 50% bases with a Phred quality score < 20) and reads with unknown nucleotides (more than 1% ambiguous residues N) using the FastQC tool (http://www.bioinformatics.babraham.ac.uk/projects/fastqc/). Analysis of the GC content distribution was performed. Transcriptome assembly was performed using Trinity r20140717 [80] and employing paired-end method. For hierarchical clustering, Corset was used (https://code.google.com/p/corset-project/). The longest cluster sequence was obtained by clustering with Corset hierarchy as unigene for subsequent analysis. The assembled unigenes were then annotated in various databases such as KEGG using KAAS (E-value < 1.0 × 10^− 10^), GO using Blast2GOv2.5 (E-value < 1.0 × 10^− 6^), PFAM using HMMER3.0 (E-value < 0.01), Swissprot using BLAST 2.6.0+ (E-value < 1.0 × 10^− 5^) and NR using BLAST 2.6.0+ (E-value < 1.0 × 10^− 5^).

The sequenced reads were compared with the unigene library using Bowtie2 [81], and the level of expression was estimated in combination with RSEM [82]. The gene expression level was determined according to the fragments per kilobase of exon per million fragments mapped (FPKM) method. The Principal component analysis was performed in R v2.3.0. The read count was normalized and EdgeR Bioconductor package [83], was used to determine the differential expression genes (DEGs) between groups with the |log2 fold change| ≥ 2 and false discovery rate (FDR) correction set at *P* < 0.05 [54]. GO enrichment analysis was performed using the topGO method based on the wallenius noncentral hypergeometric distribution with *P* < 0.05 [84]. KEGG pathway enrichment analysis of the DEGs was done using KOBAS2.0 [85]. The FDR correction was employed (*P* < 0.05) to reduce false positive prediction of enriched GO terms and KEGG pathways.

### Gene expression analysis using quantitative real PCR

In order to confirm the gene expression levels obtained from the RNA-seq, a qRT-PCR analysis was performed on RNA extracted from needle samples as described previously [86]. The qRT-PCR was conducted on a Roche Lightcyler® 480 instrument using the SYBR Green Master Mix (Vazyme, Vazyme Biotech Co. Ltd., Nanjing, China) following the manufacturer’s protocol. The gene *Actin*2 was used as internal control. Specific primer sequences for selected genes were designed with PrimerPremier 5 and are presented in Table S1.

## Supplementary Information

**Additional file 1: Table S1.** Primer sequences used for qRT-PCR in this study.

**Additional file 2: Figure S1.** GO enrichment analysis. SL0, CK0 and CK04 represent the *Suillus luteus* (SL) inoculated plants without Al stress application, SL inoculated plants with 0.4 mmol L^− 1^ Al stress application, plants non-inoculated without Al stress application and plants non-inoculated with 0.4 mmol L^− 1^ Al stress application, respectively. **Figure S2.** KEGG enrichment analysis. SL0, CK0 and CK04 represent the *Suillus luteus* (SL) inoculated plants without Al stress application, plants non-inoculated without Al stress application and plants non-inoculated with 0.4 mmol L^− 1^ Al stress application, respectively.

## Data Availability

The datasets supporting the conclusions of this article are available in the NCBI Bioproject repository, accession number: PRJNA636599. The data will be released upon publication of this manuscript.

## Abbreviations

Al: Aluminum
SL: *Suillus luteus*
ROS: Reactive oxygen species
MDA: Malondialdehyde
SOD: Superoxide dismutase
POD: Peroxidase
FPKM: Fragments per kilobase of exon per million fragments mapped
PCA: Principal component analysis
DEG: Differentially expressed genes
FDR: False discovery rate
