# Supplementary material for: Identification of candidate genes conferring tolerance to aluminum stress in Pinus massoniana inoculated with ectomycorrhizal fungus
Source: BMC Plant Biol. 2020 Nov 16;20:521. doi: 10.1186/s12870-020-02719-3 (PMC7667757; doi:10.1186/s12870-020-02719-3)

Supplementary files

Figure S1. GO enrichment analysis. SL0, CK0 and CK04 represent the *Suillus luteus* (SL) inoculated plants without Aluminum stress application, SL inoculated plants with 0.4 mmol L^{-1} Aluminum stress application, plants non inoculated without Aluminum stress application and plants non inoculated with 0.4 mmol L^{-1} Aluminum stress application, respectively.

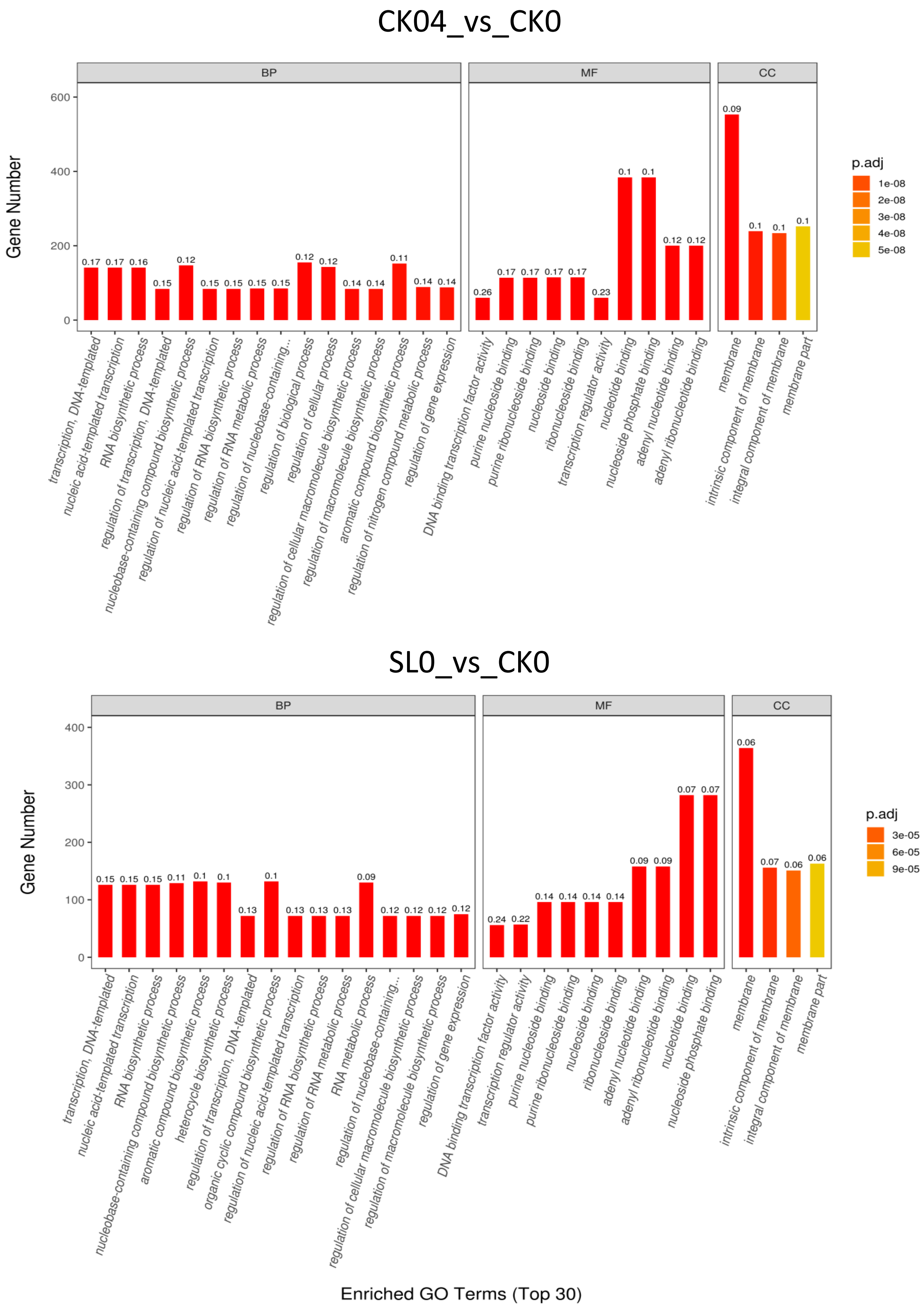

SL04_vs_CK04

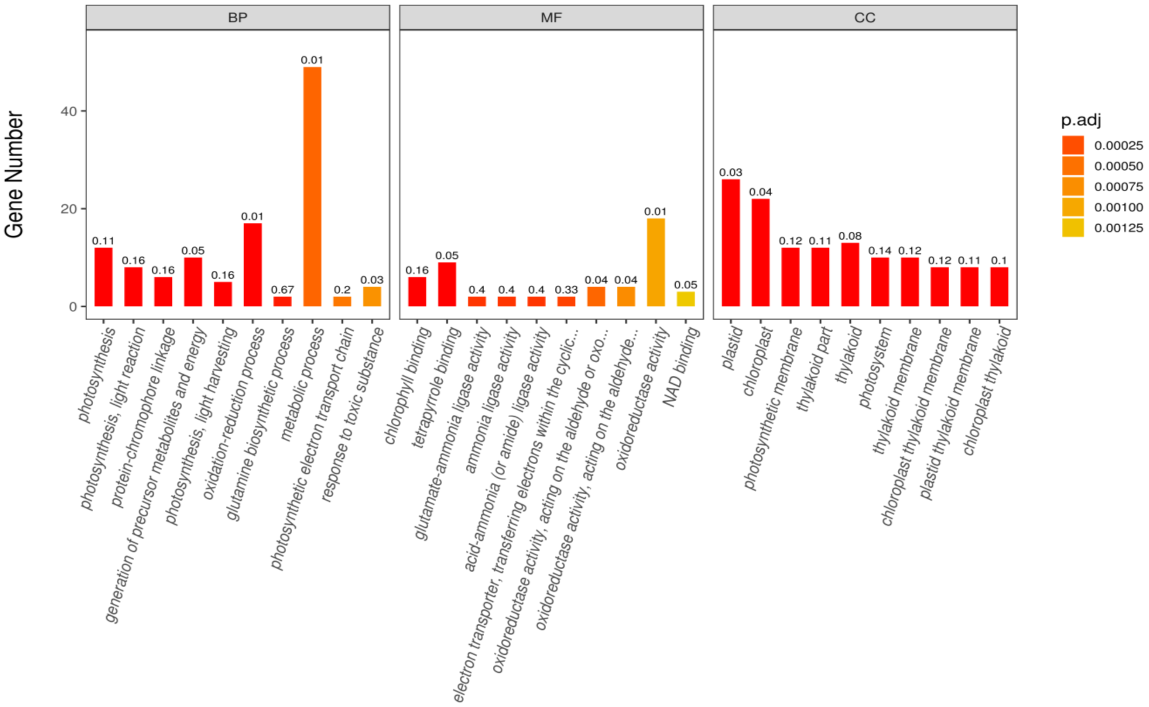

BP
MF
CC
Gene Number
p.adj
0.00025
0.00050
0.00075
0.00100
0.00125
photosynthesis
photosynthesis, light reaction
protein-chromophore linkage
generation of precursor metabolites and energy
photosynthesis, light harvesting
oxidation-reduction process
glutamine biosynthetic process
metabolic process
photosynthetic electron transport chain
response to toxic substance
chlorophyll binding
tetrapyrrole binding
glutamate-ammonia ligase activity
ammonia ligase activity
acid-ammonia (or amide) ligase activity
electron transporter, transferring electrons within the cyclic...
oxidoreductase activity, acting on the aldehyde or oxo...
oxidoreductase activity, acting on the aldehyde...
oxidoreductase activity
NAD binding
plastid
chloroplast
photosynthetic membrane
thylakoid part
thylakoid
photosystem
thylakoid membrane
chloroplast thylakoid membrane
plastid thylakoid membrane
chloroplast thylakoid

Figure S2. KEGG enrichment analysis. SL0, CK0 and CK04 represent the *Suillus luteus* (SL) inoculated plants without Aluminum stress application, plants non inoculated without Aluminum stress application and plants non inoculated with 0.4 mmol L^{-1} Aluminum stress application, respectively.

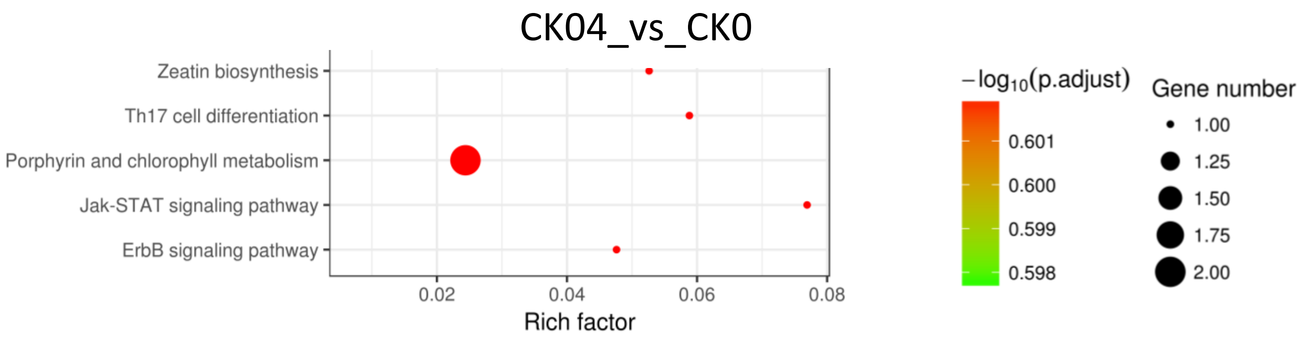

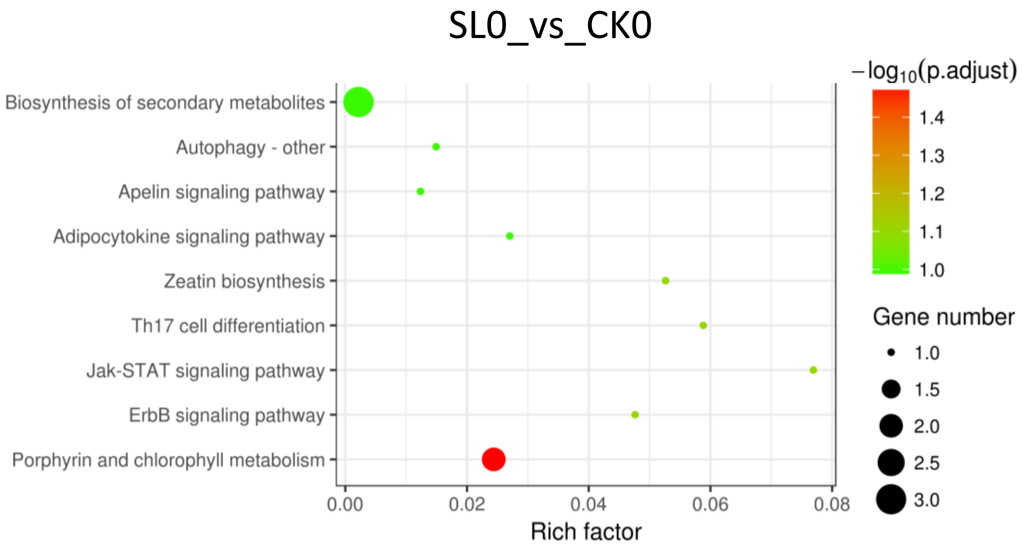

Supplement: Supplementary file 2 — Additional file 2: Figure S1. GO enrichment analysis. SL0, CK0 and CK04 represent the Suillus luteus (SL) inoculated plants without Al stress application, SL inoculated plants with 0.4 mmol L− 1 Al stress application, plants non-inoculated without Al stress application and plants non-inoculated with 0.4 mmol L− 1 Al stress application, respectively. Figure S2. KEGG enrichment analysis. SL0, CK0 and CK04 represent the Suillus luteus (SL) inoculated plants without Al stress application, plants non-inoculated without Al stress application and plants non-inoculated with 0.4 mmol L− 1 Al stress application, respectively. [file 12870_2020_2719_MOESM2_ESM.pdf]
